# Improving quality of life for a patient with fibromyalgia and their caregiver: A protocol for the application of the integrative medical service model

**DOI:** 10.1097/MD.0000000000033643

**Published:** 2023-05-05

**Authors:** Moon Joo Cheong, Chong Hyuk Chung, Chang Hoon Lee, Myeung Su Lee, Won Bae Ha, Jung Han Lee, Hyung Won Kang

**Affiliations:** a Rare Incurable Disease Integrative Medicine Treatment Laboratory, Jeonnam, Republic of Korea; b Division of Rheumatology, Department of Internal Medicine, Wonkwang University Hospital, Iksan, Republic of Korea; c Research Center of Traditional Korean Medicine & Department of Korean Medicine Rehabilitation, College of Korean Medicine, Wonkwang University, Iksan, Republic of Korea; d Department of Neuropsychiatry of Korean Medicine, College of Korean Medicine, Wonkwang University, Iksan, Republic of Korea.

**Keywords:** caregivers, feedback, fibromyalgia, integrative healthcare service, protocol, quality of life

## Abstract

Patients with incurable diseases experience difficulty carrying out activities of daily living and rely on caregivers. Caregivers of patients with fibromyalgia (FM) are unable to understand the extent of the patients’ suffering because the pain sites are invisible. To address this problem, this study will apply an integrative healthcare service model to a single FM case to manage pain and enhance the quality of life and, subsequently, gather feedback from different sources regarding the treatment. This paper presents the study protocol.

**Methods:**

We will conduct an observational study to gather quantitative and qualitative feedback from various perspectives regarding the application of an integrative healthcare service program for FM patients developed in Korea for an FM patient–caregiver pair. The program will comprise eight 100-minute weekly sessions, during which integrative services that combine Western and Oriental medicines (Korean traditional medicine) will be provided to enhance pain management and quality of life. The feedback collected after each session will be reflected in the next session’ content.

**Results:**

The results will comprise the feedback from the patient and caregiver in accordance with revisions made to the program.

**Conclusions:**

The results will provide basic data for optimizing an integrative healthcare service system in Korea for patients suffering from chronic pain owing to diseases such as FM.

## 1. Introduction

The 4 major diseases – heart disease, brain disease, cancer, and rare and incurable diseases – are chronic, severe, and geriatric diseases; thus, they must be approached from an integrative healthcare service perspective rather than a general therapeutic perspective.^[1]^ Chronic diseases require continuous treatment, leaving caregivers with financial, psychological, and physical burdens.^[2]^ Unfortunately, the current healthcare system fails to provide sufficient methods or services to address this burden.^[3]^ Nevertheless, medical experts agree that diverse forms of healthcare services are required for patients with major severe and geriatric chronic diseases and their caregivers.^[4]^

Against this background, we conducted a national study in Korea to determine a method and framework for developing an integrative healthcare service model for patients with the 4 major severe and geriatric chronic diseases.^[5]^ However, empirical feedback from patients and caregivers remains necessary to improve the model’s accessibility in clinical settings and to develop it into a well-established practical model for patients and their caregivers. Accordingly, we plan to conduct a case study to uncover insights for improving the integrative healthcare service model. This paper outlines the protocol for this future study. Specifically, we intend to conduct a case study with a patient with fibromyalgia (FM) and the caregiver, applying an integrative healthcare service model specifically developed to help patients with FM manage their pain and improve the quality of life (QoL) of both the patient and their caregivers. Patients with FM deal with constant excruciating pain that prevents them from performing activities of daily living and severely deteriorates their QoL. Such adverse impacts also lead to conflicts with caregivers as well as drug misuse and abuse.^[6]^ Nevertheless, the on-site implementation of appropriate management plans remains difficult.^[7]^ To resolve this, we aim to collect weekly feedback from the patient and the caregiver to explore the effects and possibilities of our model and identify the necessary revisions therein.

## 2. Materials and methods

### 2.1. Methodology

This observational study will apply the integrative healthcare service program developed in Korea for FM patients’ pain management and QoL enhancement for the patient and their caregiver (the patient–caregiver pair). We will collect quantitative and qualitative data through participant feedback to determine best practices for the practical application of the program. Before obtaining consent, the research manager will explain the study to the participants, including the procedure and the compensation they will receive, and advise them that they can withdraw from the study at any time. The researchers will collect, share, and maintain personal information about potential and registered participants. To protect confidentiality before, during, and after the trial, the research manager will store all data on their personal computer and act in accordance with institutional review board guidelines. All data will be discarded 3 years after study completion.

### 2.2. Participants

#### 2.2.1. Patient.

The inclusion criteria for the patient will be as follows: aged 20 to 65 years; clinically diagnosed by a specialist, a Widespread Pain Index (WPI) ≥ 7, Symptom Severity Scale (SSS) score ≥ 5, and duration of chronic pain ≥ 3 months according to the criteria set by the American College of Rheumatology (ACR) in 2016 or aWPI 3 to 6, SSS score ≥ 9, and duration of chronic pain ≥ 3 months according to the criteria set by the ACR in 2016;^[8]^ capable of reading and completing the questionnaire; and able to understand the purpose of this research and willing to participate in the study.

The exclusion criteria for the patient will be as follows: a history of major psychiatric disorders, such as psychiatric symptoms listed in the DSM-5 (i.e., schizophrenia spectrum disorder, delusional disorder, bipolar disorder, and alcohol/substance use disorder), a history of chronic diseases that may affect the research results, participation in other clinical trials within the past month, incapable of typical communication owing to dementia or mild cognitive impairment, diagnosed with a somatoform disorder, in which pain is the primary complaint with no clear organic cause and psychological problems as common features, problems communicating and a limited ability to read and complete the questionnaire, and judged incapable of participating in the study by the principal researcher based on medical conditions.

#### 2.2.2. Caregivers.

The term “caregiver” refers to an adult member of a family or a trusted person who assists the patient in performing activities of daily living. The inclusion criteria for the caregiver will be as follows: aged 20 to 65 years, designated as a caregiver by the enrolled patient, and willing to participate in the study. Caregivers will be excluded if the principal researcher finds them incapable of participating in the study owing to communication problems associated with serious psychiatric conditions, intellectual disabilities, mood disorders, or other cognitive problems.

### 2.3. Sample size

The patient–caregiver pair will be provided with healthcare services, after which they will be required to give feedback. As per Boddy’s^[9]^ recommendation that a case study’s sample size should be determined according to the situation and partly depends on the scientific paradigm according to which the investigation is conducted, 2 samples – a patient and a caregiver, together comprising a patient–caregiver pair – were selected.

### 2.4. Integrative healthcare service model

This study will use the “integrative healthcare service model for managing pain and enhancing the QoL in patients with FM.” The model contains the following features. Among patients who visit a healthcare facility, those with FM are diagnosed and treated by a joint medical staff of Western and Korean traditional medicine specific to the Korean healthcare service system. Additional integrative treatment options consist of a range of complementary and alternative services for physical pain management interventions derived from literature and expert opinions. Supplemental and integrative healthcare services are provided to both patients and caregivers. Caregivers receive information on the characteristics of the patient’s syndrome from medical staff and the coordinator in charge of integrative healthcare services. Owing to the invisibility of pain sites in FM, others may doubt the patient’s diagnosis; therefore, the medical staff starts by explaining the syndrome to caregivers. Next, caregivers are shown a video that depicts the lives of patients with FM. Additionally, patients and caregivers engage in collaborative programs (e.g., art, horticultural, and exercise therapies) designed to improve patient–caregiver relationships and help the caregiver better understand the patient. Finally, caregivers are provided with information on financial aid – including insurance – and healthcare provider and community networks for daily living activity support after the program’s completion.

### 2.5. Integrative healthcare service program

#### 2.5.1. Program composition.

The program is based on the existing integrative healthcare service model for managing pain and improving QoL in patients with FM and was developed as part of the Ministry of Health and Welfare’s integrated healthcare support project. In this study, the research team will continuously improve the program based on the feedback gathered during each session and in consultation with the medical staff to ensure that the program’s changes are conducive to clinical application.

##### 2.5.1.1. Healthcare services

The patient is consulted about the current stage of the syndrome, prescriptions for medications, and pain injections. Next, Oriental medicine consultations and services are provided, such as acupuncture, Chuna therapy, and physiotherapy.

##### 2.5.1.2. Integrative services

Integrative patient services include breathing meditations, exercise, and treatments or interventions that apply various media for pain management and emotional control. The breathing meditation program is guided by an expert or self-directed according to the instructions of a 5-minute video clip. The exercise program focuses on pain management, and when conducted together with the caregiver, materials (i.e., media) for later use can be provided; this program offers various experiences to improve mind–body health and QoL for the patient and caregiver. Multifaceted media interventions immerse the patient in an activity as a way to distract the patient from feeling pain. If a program involves the caregiver, it increases their understanding of the syndrome and the patient, which can improve their relationship. Each part of the program is administered by a corresponding expert or specialist.

##### 2.5.1.3. Special feature

Program details will be adjusted every week according to opinions and feedback from the experts, patients, and caregivers.

### 2.6. Program implementation plan

Consultations and medical treatments will be provided in the presence of medical staff in safe places in Western and Oriental hospitals across 8 weekly 100-minute sessions. Table 1 below presents an example of this program.

**Table 1:** Integrative healthcare service program for a fibromyalgia patient–caregiver pair.

Session	Fibromyalgia program: Pain management and QoL enhancement						Duration
1(Aug 5)	P	Program orientation and filling out the questionnaire (20 min)	Breathing meditation (5 min)	Aromatherapy massage (40 min)		Questionnaire + opinion gathering (10 min)	≤100 min
	C			A full psychological test battery (30 min)	Physiotherapy: Healing Center (10 min)	Questionnaire + opinion gathering (10 min)	
2 (Aug 12)	P	OM treatment	Breathing meditation (5 min)	Yoga program (P + C) (50 min)	Physiotherapy: OM Division	Gathering P opinions (10 min)	≤100 min
	C	Psychological test interpretation			Physiotherapy: Healing Center (30 min)	Gathering C opinions (10 min)	
3 (Aug 16)	P	OM treatment	Breathing meditation (5 min)	Art therapy (P + C) (50 min)		Gathering P opinions (10 min)	≤100 min
	C	Explanation of FM				gathering C opinions (10 min)	
4 (Aug 23)	P	OM treatment	Horticultural therapy: meeting room (40 min)			Questionnaire + opinion gathering (10 min)	≤100 min
	C	Physiotherapy for the C (WM)				Questionnaire + opinion gathering meeting room (10 min)	
5 (Aug 29)	P	Reception in Iksan Hospital (C waiting in hospital)	Medication instruction: Pharmacist - nutrition and medication education (15–20 min)	Consultation with the director (9:30 am). Treatment: physiotherapy		Gathering P opinions (10 min)	≤100 min
	C			FM explained by the director of the OM hospital while C is getting physiotherapy		Gathering C opinions (10 min)	
6 (Sep 5)	P	Consultation and treatment (OM) (≤20 min)	Breathing meditation (5 min)	Kinesio pain management taping: meeting room (P + C)	Health food medication instruction: OM hospital (100 min)	Gathering P opinions (10 min)	≤100 min
	C	Physiotherapy (OM)				Gathering C opinions (10 min)	
7 (Sep 14)	P	Treatment and treatment (OM) (≤20 min)	Breathing meditation (5 min)	Laughter therapy or singing therapy: meeting room (40 min)		Gathering P opinions (10 min)	≤100 min
	C	Physiotherapy (OM)				Gathering C opinions (10 min)	
8 (Sep 23)	P	Consultation and treatment (OM) (≤20 min)	Breathing meditation (5 min)	Art Therapy II: gathering P and C opinions (50 min)	P psychological test interpretation	Final questionnaire and collection of P opinions	≤100 min
	C	Physiotherapy (OM)			Final questionnaire and collection of C opinions		

C = caregiver, FM = fibromyalgia, OM = Oriental medicine, P = patient, WM = Western medicine.

### 2.7. Assessment items

#### 2.7.1. Sociodemographic and health-related characteristics of the patient and caregiver.

This study will consider the following participant sociodemographic and health-related variables: sex, age, marital status, educational level, health insurance coverage type, assumption of personal care costs, primary caregiver, duration of illness, the person who pays their hospital bills, religion, occupation, self-rated economic status, age at diagnosis, current medication(s), underlying diseases, participation in exercise (yes/no; if yes, exercise types), and the measures taken to reduce pain.

#### 2.7.2. Primary outcome for the patient and caregiver.

##### 2.7.2.1. Health-related quality of life (HRQoL) assessment tool

The HRQoL measure will be the EuroQoL-5 Dimension 5-Level (EQ-5D-5L). The EQ-5D was launched by the EuroQol Group in 1987. The Korean EuroQol-5 dimension is a multidimensional preference-based HRQoL measure that assesses HRQoL in light of utility. The EQ-5D-5L was launched by the EuroQol Group as a new QoL measurement tool, with the 3 domains of the EQ-5D-3L extended to 5 to compensate for its shortcomings. While the EQ-5D-3L describes 243 health states, the EQ-5D-5L describes 3125 health states, reducing the ceiling effect of the EQ-5D-3L and increasing the reliability and sensitivity of the instrument.^[10]^

The general health status measure will be the EuroQoL visual analog scale.^[11]^ The EuroQoL visual analog scale is a health state rating scale that records the self-rated health state on a 20-cm-long vertical visual analog scale. The scale assigns top-to-bottom numeric values ranging from 100 (best imaginable health state) to 0 (worst imaginable health state). Scores order health outcomes and preferences.

#### 2.7.3. Secondary outcome.

##### 2.7.3.1. Patient outcomes

The first patient outcome is pain assessment. The following instruments will be used to assess pain, pain severity, and impairment of daily living activities. First, the extent of FM pain will be measured using the WPI developed by Wolfe et al^[12]^ and approved by the ACR as the new preliminary diagnostic criteria in 2010. This tool is widely used in rheumatology to diagnose FM and develop the prognosis. A WPI score is obtained by adding up the number of checked items on the checklist or on the front and back images of a human model mannequin. The total score ranges from 0 to 19, with a higher score indicating a higher number of pain sites. Second, the severity of FM symptoms will be measured using the SSS developed by Wolfe et al^[12]^ and approved by ACR as the new preliminary diagnostic criteria in 2010. This tool is widely used in rheumatology to diagnose FM and develop a prognosis. The total score ranges from 0 to 12, with a higher score indicating a higher symptom severity. Third, the impact of FM on the patient will be measured using the Fibromyalgia Impact Questionnaire developed by Wolfe et al^[12]^ and approved by ACR as the new preliminary diagnostic criteria in 2010. The symptom assessment items of this tool, which are measured on a numeric rating scale, include the degree of physical impairment; number of days the patient felt “good”; degree of disruption, fatigue, waking up refreshed or unrefreshed; stiffness; anxiety; and depression. The total score ranges from 0 to 100, with higher scores indicating more severe FM. At the time of development, the test-retest reliability (r) of the physical impairment scale ranged from 0.80 to 0.96, and the reliability (Cronbach α) of the Fibromyalgia Impact Questionnaire was 0.88. Fourth, the Numeric Pain Rating Scale (NPRS) will be applied to determine the patient’s pain intensity. The NPRS is an easy-to-use pain intensity rating scale rated on an 11-point scale (0 = no pain, 10 = worst pain).^[12]^ Specifically, this study will apply the Triple NPRS to evaluate the patient’s pain intensity; this approach involves calculating the overall average by receiving the patient’s current, highest, and lowest pain scores for the past 24 hours.^[13]^ The intraclass correlation coefficient for the test-retest reliability of the Triple NPRS was reported to range from 0.61 to 0.77, with the minimal clinically important difference calculated at 2 points.^[14]^

Meanwhile, the Pittsburgh Sleep Quality Index was used to assess sleep quality. The Pittsburgh Sleep Quality Index is a 19-item self-report questionnaire developed by Bysse et al^[15]^ The index determines sleep quality by measuring 7 components of sleep: sleep latency, subjective sleep quality, sleep duration, habitual sleep efficiency, sleep disturbance, use of sleeping medication, and daytime dysfunction. Additionally, 5 items will be collected from individuals living in the same household; these data points are not considered in the score but are used to obtain clinical information. Each item is rated on a 4-point scale ranging from 0 to 3, with the total score ranging from 0 to 21. Higher scores indicate poorer sleep quality; a score of 5 or higher indicates a sleep problem.

##### 2.7.3.2. Caregiver outcomes

Caregiver outcomes include burden and happiness levels. First, the caregiver burden will be measured using the Korean version of the Caregiver Burden Inventory developed by Novak and Guest^[16]^ and translated and revised by Cheong.^[17]^ The Korean version of the Caregiver Burden Inventory divides the caregiver burden into 6 domains: time-dependent burden (items 1–5), development and achievement burden (items 6–10), physical burden (items 11–14), social burden (items 15–19), emotional burden (items 20–24), and financial burden (items 25–29). Each of the 29 items is rated on a 5-point scale (1 = strongly disagree, 5 = strongly agree), with the total score ranging from 29 to 145. Higher scores indicate a greater caregiver burden. Meanwhile, the caregiver’s level of happiness will be measured using the Oxford Happiness Questionnaire developed by Hills and Argyle^[18]^ and translated by Choi and Lee.^[19]^ This 29-item scale measures the degree of personal happiness in the domains of self-control, positive emotions, and confidence. Each item is rated on a 6-point scale (1 = strongly disagree, 6 = strongly agree). Negative questions are reverse-scored. Higher scores indicate a higher level of happiness. The reliability (Cronbach α) of the scale was 0.90 in the study by Choi and Lee^[19]^ and 0.93 in a study by Cho (2016).

##### 2.7.3.3. Patient–caregiver outcome

The patient–caregiver outcomes include emotional and mental states. The Core Seven-Emotions Inventory-short form (16) will be used to measure emotional state. The Core Seven-Emotions Inventory-short form is a 28-item scale consisting of 7 domains representing basic human emotions in oriental medicine: joy (4 items), anger (4 items), thinking (4 items), anxiety (4 items), sorrow (4 items), fear (4 items), and fright (4 items). Each item is rated on a 5-point Likert scale, whereby the scores of 6 emotions excluding joy surpassing the T score (M = 50, SD = 10) are considered in the at-risk range – the higher the score, the higher the risk. Specifically, the T score cutoff points are 55 to 60 for the caution group, 61 to 65 for the risk group, and ≥66 for the high-risk group. By contrast, lower joy scores indicate a higher risk: 40 to 45 = caution group, 35 to 39 = risk group, and ≤35 = high-risk group. Meanwhile, mentalizing the rooms of mind (MRM) will be used to measure mental state. The MRM is a projective test and a Mindfulness and Loving Beingness psychotherapy technique designed to help participants visualize and realize their mental states. It encourages the individual to describe their state of mind in terms of interactional relationships, and a psychological counselor can further help the individual to cope with any vague states of mind by facilitating introspection. This is a useful tool with high clinical utility in the evaluation and improvement of mental states.

### 2.8. Assessment plan

#### 2.8.1. Participant assessment.

Participants will be evaluated on the assessment items twice: at baseline (pre-assessment; Session 1) and after the intervention (post-assessment, Session 8). Observational program assessment will be performed during each session using the MRM tool and patient and caregiver feedback. Research details are presented in Table 1, and the assessment plan is outlined in Table 2.

**Table 2:** Research flow and assessment plan for the patient with fibromyalgia and the caregiver.

	Period			Screening (consent, enrollment)	Pre-assessment (Session 1)	Post-assessment (Session 8)
Results of inclusion/exclusion criteria				○		
Written consent form (reading and signing)				○		
Patient	Sociodemographic survey				○	
	Psychological testing				○	
	Primary outcome	HRQoL assessment scales	EQ-5D-5L		○	○
			EQ-VAS			
	Secondary outcome	Instruments for assessing pain, severity, and impairment of daily living activities			○	○
		Numeric Pain Rating Scale			○	○
		CSEI-s			○	○
		Pittsburgh Sleep Quality Index			○	○
Caregiver	Sociodemographic survey				○	
	Psychological testing				○	
	Primary outcome	HRQoL assessment scales	EQ-5D-5L		○	○
			EQ-VAS			
	Secondary outcome	Caregiver Burden Inventory			○	○
		CSEI-s			○	○
		Oxford Happiness Questionnaire			○	○

CSEI-s = Core Seven-Emotions Inventory-short form, EQ-5D-5L = EuroQoL-5 Dimension 5-Level, EQ-VAS = EuroQoL visual analog scale, HRQoL = health-related quality of life, QoL = quality of life.

#### 2.8.2. Research team’s assessment (session report on the expert and participant feedback).

After each session, the researchers who guided the patient and the caregiver through their respective programs will listen to and record their feedback. Next, the principal researcher, the researchers who gathered the patient’s and caregiver’s feedback, and the experts involved in each session will discuss the feedback as it relates to the program administered at each session. Specifically, they will review opinions on the shortcomings, develop comprehensive plans to implement improvements and consider anticipated costs to implement these improvements in future clinical applications. Finally, the feedback contents will be reflected in the program details for the next session to continuously improve the program during the study period.

### 2.9. Data analysis

Assessment data will be analyzed using quantitative and qualitative methods. Regarding the quantitative analysis, the characteristics of the questionnaires for patients and caregivers regarding pain management and QoL enhancement in patients with FM will be analyzed. Data for inferential statistical analysis will be gathered twice: at baseline prior to Session 1 and after the intervention at the end of Session 8. Exploratory result analysis will be performed using the paired sample chi-square test for each questionnaire completed at baseline and after the program. Meanwhile, the patient and caregiver feedback collected after each session and the results of MRM testing for projected emotions will be qualitatively analyzed.

### 2.10. Ethical considerations

Designed as an observational study, this study is unlikely to involve any risks other than those that may occur in the general treatment process. However, since mental and psychological risks cannot be ruled out during an interview, a therapist, an FM specialist in charge of the patient, and the caregiver will stand by to intervene when necessary.

The program of this study will be completed within a 100-minute window, with a 10-minute break after 60 minutes to mediate or prevent physical and mental fatigue. Additionally, participants can request a break whenever needed during the program.

The information and data obtained from the research participants will be protected and used under absolute anonymity guarantees to ensure that their identities cannot be disclosed under any circumstances. Additionally, when the results of this study are published, caution will be taken to ensure that participants’ personal information is not disclosed anywhere in the published article. All data collected and obtained during the study shall be used exclusively for research purposes and report writing. Participants’ data will be stored securely in the principal researcher’s archives to prevent loss or theft. After a 3-year retention period, all data will be destroyed and discarded.

## 3. Discussion

FM is a pain syndrome that affects soft tissues, such as tendons, ligaments, fascia, muscles, and adipose tissue, causing chronic musculoskeletal pain, stiffness, paresthesia, sleep disturbances, and fatigue throughout the body, with tender point sites appearing throughout the body.^[20,21]^ The number of patients with FM in Korea exceeds 20,000, accounting for 1 to 2% of the total population. A US epidemiological study also estimated the prevalence of FM in the US at 1 to 2% of the total population, which suggests that FM is no longer a rare disease.^[22,23]^ However, FM remains an incurable disease with an unclear cause and ineffective treatment options. Pain from FM is so strong that patients call it the “worst” among rheumatic diseases.^[24]^ In the past, with no proper treatment options, patients relied only on medication. With the treatment paradigm evolving, recent trends are reducing patients’ pain and enhancing their QoL.^[23]^ In this context, this study will apply the FM patients’ pain management and QoL enhancement model developed for the application of integrative healthcare services to patients with the 4 major severe and geriatric diseases. In doing so, this study will systematically integrate interventions into a healthcare service system for patients with chronic pain and gather opinions from patients and caregivers about the effectiveness of the interventions.

The universality of this study’s data is limited by the fact that the study will only involve a single patient–caregiver pair; thus, varied feedback will not be collected. However, the protocol can be used in future studies on other patient–caregiver pairs, in which more emphasis can be applied to collecting more in-depth opinions by obtaining detailed feedback from the patient and caregiver after each session. Nevertheless, using the single-case protocol provides flexibility that multiple-case protocols cannot; thus, this protocol can be leveraged to modify and improve the program as it allows for individualized improvement based on the feedback received at each session. This offers the advantage of verifying which program is most satisfactory and useful for QoL enhancement from the patient’s and caregiver’s perspectives. The results of this study are expected to serve as the basis for revising the integrative healthcare service model into an optimized patient-centered model. The revised model will be used for future clinical pathway development research to make it applicable in clinical settings in every healthcare facility.

## Author contributions

**Conceptualization:** Moon Joo Cheong, Chong Hyuk Chung, Hyung Won Kang.

**Data curation:** Moon Joo Cheong, Chong Hyuk Chung, Hyung Won Kang.

**Formal analysis:** Moon Joo Cheong.

**Funding acquisition:** Hyung Won Kang.

**Investigation:** Moon Joo Cheong.

**Methodology:** Moon Joo Cheong.

**Project administration:** Moon Joo Cheong.

**Supervision:** Hyung Won Kang.

**Writing – original draft:** Moon Joo Cheong.

**Writing – review & editing:** Moon Joo Cheong, Chong Hyuk Chung, Chang Hoon Lee, Myeung Su Lee, Won Bae Ha, Jung Han Lee, Hyung Won Kang.
